# Supplementary material for: A class-contrastive human-interpretable machine learning approach to predict mortality in severe mental illness
Source: NPJ Schizophr. 2021 Dec 8;7:60. doi: 10.1038/s41537-021-00191-y (PMC8654849; doi:10.1038/s41537-021-00191-y)
Supplement: Supplementary file 1 — Supplementary Information [file 41537_2021_191_MOESM1_ESM.pdf]

A human-interpretable machine learning approach to predict mortality in severe mental illness: Supplementary Information

Soumya Banerjee [1*], Pietro Liò[2], Peter B. Jones[1,3], Rudolf N. Cardinal[1,3]

1 Department of Psychiatry, University of Cambridge, UK

2 Department of Computer Science and Technology, University of Cambridge, UK

3 Cambridgeshire and Peterborough NHS Foundation Trust, UK

∗ Corresponding author E-mail: sb2333@cam.ac.uk

Supplementary Information

Sensitivity analysis

Additional analysis

For the ML model (random forest built on features from the autoencoder), we performed additional sensitivity analysis. We repeated the stochastic process of splitting the data into training and test sets and performing cross-validation 10 times. We then performed stability analysis of heatmaps. We generated heatmaps for each of the 10 iterations mentioned above. We show a representative heatmap in Supp. Fig. 1.

Because of heterogeneity in training data and correlations across features, reproducibility of heatmaps is a challenge. We acknowledge this limitation. We show a representative example in Supp. Fig. 1. This heatmap is consistent with previous results (Fig. 6), with the exception that it shows use or prescription of SGA is associated with an increased probability of mortality (Supp. Fig. 1, shown by arrow on bottom left corner). This is not consistent with previous results from the logistic regression model and survival analysis (Fig. 4 and Fig. 3). Reconciling these results will require additional analysis in an independent cohort with more patients.

Consistent with the previous results (Fig. 6), this new heatmap (Supp. Fig. 1) also shows the counter-intuitive result that for some patients with respiratory disease or Alzheimer's disease, the model predicts a lower risk of death.

Our results suggest that the class-contrastive approach is sensitive to the training data and any imbalances in features (e.g. a particular binary feature may be 0 for 100 patients and 1 for 10 patients).

Correlations across features may also help explain these counter-intuitive results.

We also used the following models to predict mortality: 1) a random forest model operating on the original features (95% CI of AUC: [0.71, 0.79]), 2) performing PCA on the original features and using these reduced dimensions as features to a random forest model (95% CI of AUC: [0.51, 0.76]) and logistic regression model (95% CI of AUC: [0.52, 0.77]), and 3) L_1 regularized logistic regression model using the original features (95% CI of AUC: [0.72, 0.74]). We performed PCA on the original input features. The top 10 principal components were then used as input to a logistic regression model and (independently) a random forest model.

For the L_1 regularized logistic regression model, we optimized the regularization hyperparameter as described before. Briefly, we split the data into training set (50%), validation set (25%) and test set (25%). We trained the model on the training set. We carried out cross-validation on the validation set. The regularization parameter for an L_1 penalized logistic regression model is then selected. This final model is then evaluated on the test set. This process of splitting the data (into training, validation and test sets), training the model and performing cross-validation is repeated 10 times.

Finally, we also fit a L_1 regularized logistic regression model where age was divided by 100 (instead of being scaled by subtracting the mean and dividing by the standard deviation). This model had similar predictive power compared to a model where age was standardized (95% CI of AUC [0.69, 0.73]). Hence we use the standardization method throughout for the age variable (subtracting the mean and dividing by the standard deviation).

Our aim is not to exhaustively compare all possible statistical models but merely briefly survey and analyse some techniques. Our aim is to apply class-contrastive analysis to a few machine learning models and show that in some scenarios the model predictions can be explained. We note that our aim is not to demonstrate that machine learning models can perform better than others.

Our objective is not to show that a particular ML algorithm is better but to show that ML approaches can be made interpretable in some scenarios using class-contrastive reasoning. We show a practical demonstration on a clinical dataset in a disease of public health relevance.

Logistic regression models with interaction effects

Our deep learning models emphasize combinations of different features. Hence, as a very simple approximation, we also fit a more logistic regression model with interaction effects and main effects.

We fit a logistic regression model with main effects and an interaction term to account for comorbidities: dementia and cardiovascular disease (Supp. Fig. 2).

The model, in R notation, was as follows:

Death $\sim$ dementia * cardiovascular + age + dementia + delirium + abuse_alcohol_drugs + specific_personality_disorder + respiratory + cardiovascular + diabetes + self_harm + lack_family_support + personal_risk_factors + SGA + antidepressant + suicide_attempt + dementia_drug + antimanic_drug + thyroid + FGA + diuretic + anti_hypertensive + aspirin

We also fit a logistic regression model where age interacts with all other features (Supp. Fig. 3).

The model is:

Death $\sim$ age + dementia + delirium + abuse_alcohol_drugs + specific_personality_disorder + respiratory + cardiovascular + diabetes + self_harm + lack_family_support + personal_risk_factors + SGA + antidepressant + suicide_attempt + dementia_drug + antimanic_drug + thyroid + FGA + diuretic + anti_hypertensive + aspirin + age*dementia + age*delirium + age*abuse_alcohol_drugs + age*specific_personality_disorder + age*respiratory + age*cardiovascular + age*diabetes + age*self_harm + age*lack_family_support + age*personal_risk_factors + age*SGA + age*antidepressant + age*suicide_attempt + age*dementia_drug + age*antimanic_drug + age*thyroid + age*FGA + age*diuretic + age*anti_hypertensive + age*aspirin

Supplementary Figure Legends

Supp. Figure 1. Sensitivity analysis for class contrastive heatmap for the deep learning model. Visualization of the amount of change predicted in the probability of death by setting a particular feature to 1 versus 0. Predictions are made on the test set using a random forest model built on top of the autoencoder. Columns represent patients and rows represent features. The arrows at the bottom right indicate counter-intuitive examples. If these patients had a respiratory diseases or Alzheimer's disease, the model predicts low risk of death. The arrow at the bottom left indicates a group of patients on SGA who are predicted to have high risk of death. This is inconsistent with analysis from the logistic regression and survival models. The heatmap also shows a hierarchical clustering dendrogram which is performed using an Euclidean distance metric and complete linkage. We note that even though we cluster the features (columns) we do not aim to imply any similarity between them.

Supp. Figure 2. A logistic regression model with main effects and an interaction effect between dementia and cardiovascular disease. Log odds ratio for each feature from a logistic regression model for predicting mortality in patients with schizophrenia. The logistic regression model has main effects and an interaction between dementia and cardiovascular disease. Shown are confidence intervals and statistical significance (filled dark circles: p-value < 0.05, open circles: not significant).

Supp. Figure 3. Logistic regression model with main effects and all pairwise interactions with age. Log odds ratio for each feature from a logistic regression model for predicting mortality in patients with schizophrenia. The logistic regression model has main effects and all pairwise interactions with age. Shown are confidence intervals and statistical significance (filled dark circles: p-value < 0.05, open circles: not significant).

Supplementary Figures

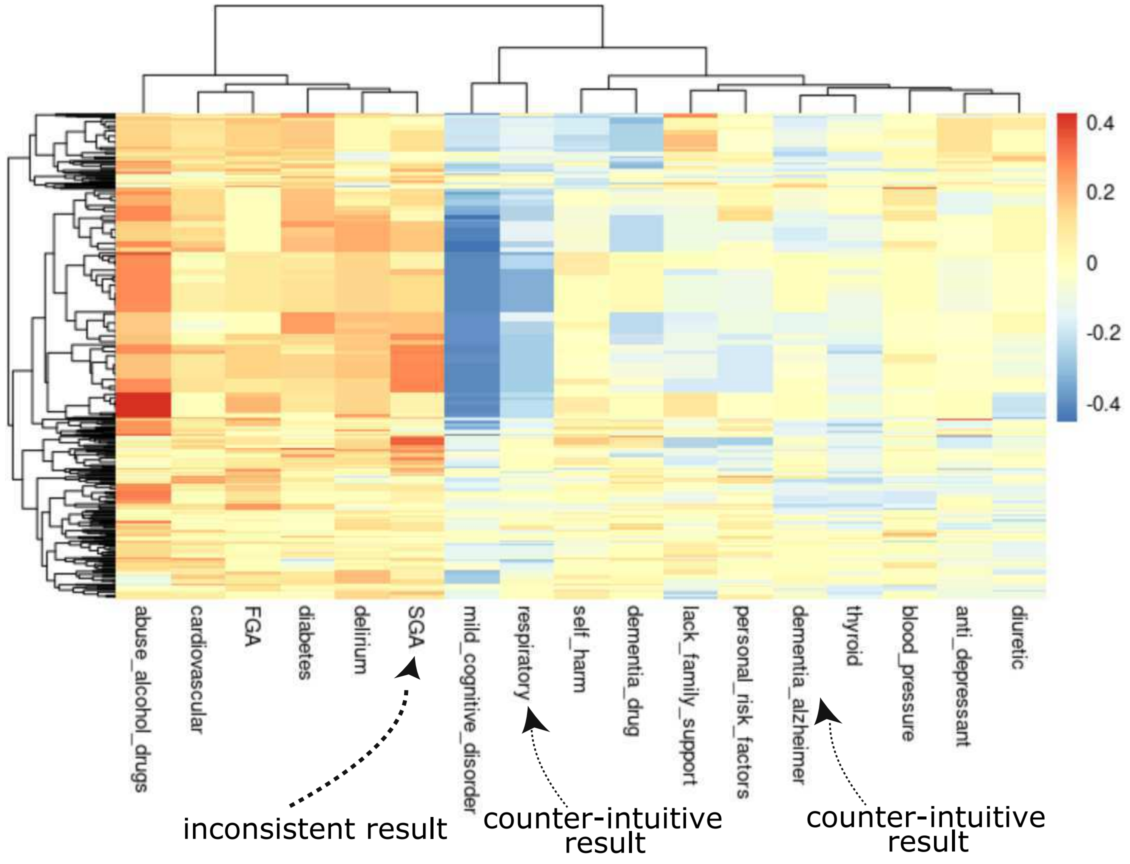

Supplementary Figure 1. Sensitivity analysis for class contrastive heatmap for the deep learning model. Visualization of the amount of change predicted in the probability of death by setting a particular feature to 1 versus 0. Predictions are made on the test set using a random forest model built on top of the autoencoder. Columns represent patients and rows represent features. The arrows at the bottom right indicate counter-intuitive examples. If these patients had a respiratory diseases or Alzheimer's disease, the model predicts low risk of death. The arrow at the bottom left indicates a group of patients on SGA who are predicted to have high risk of death. This is inconsistent with analysis from the logistic regression and survival models. The heatmap also shows a hierarchical clustering dendrogram which is performed using an Euclidean distance metric and complete linkage. We note that even though we cluster the features (columns) we do not aim to imply any similarity between them.

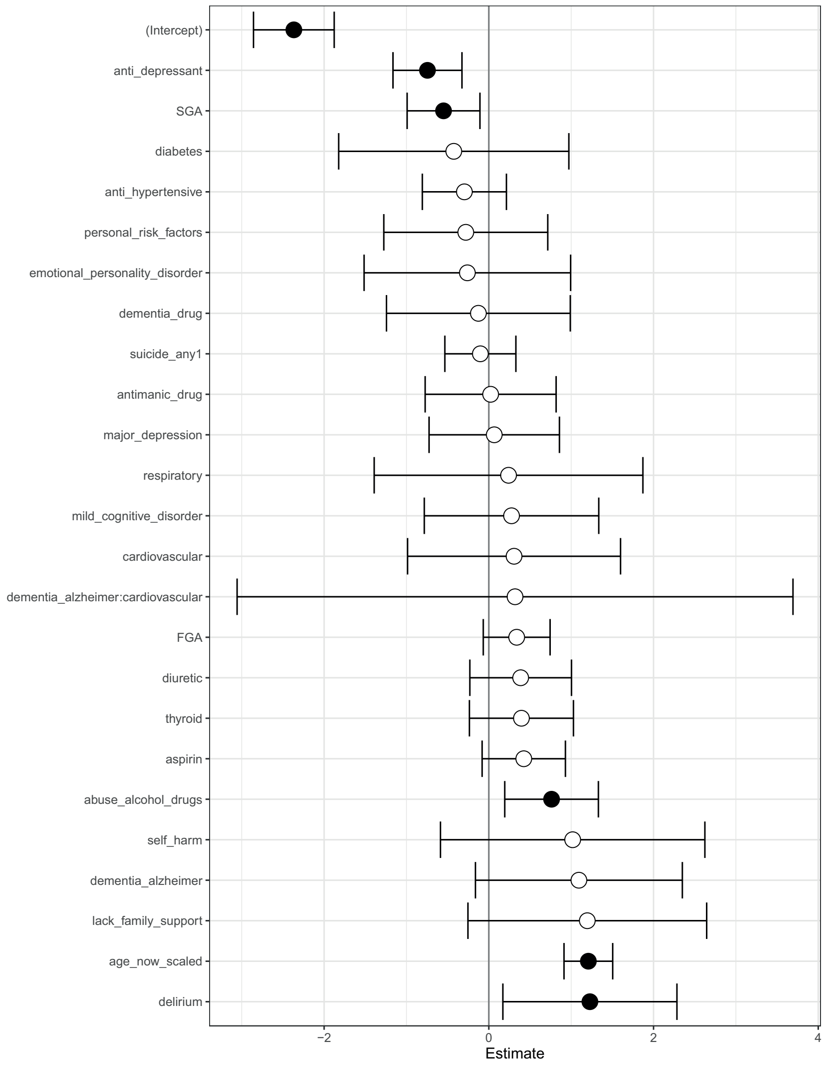

Supplementary Figure 2. Logistic regression model with main effects and an interaction effect between dementia and cardiovascular disease. Log odds ratio for each feature from a logistic regression model for predicting mortality in patients with schizophrenia. The logistic regression model has main effects and an interaction between dementia and cardiovascular disease. Shown are confidence intervals and statistical significance (filled dark circles: p-value < 0.05, open circles: not significant).

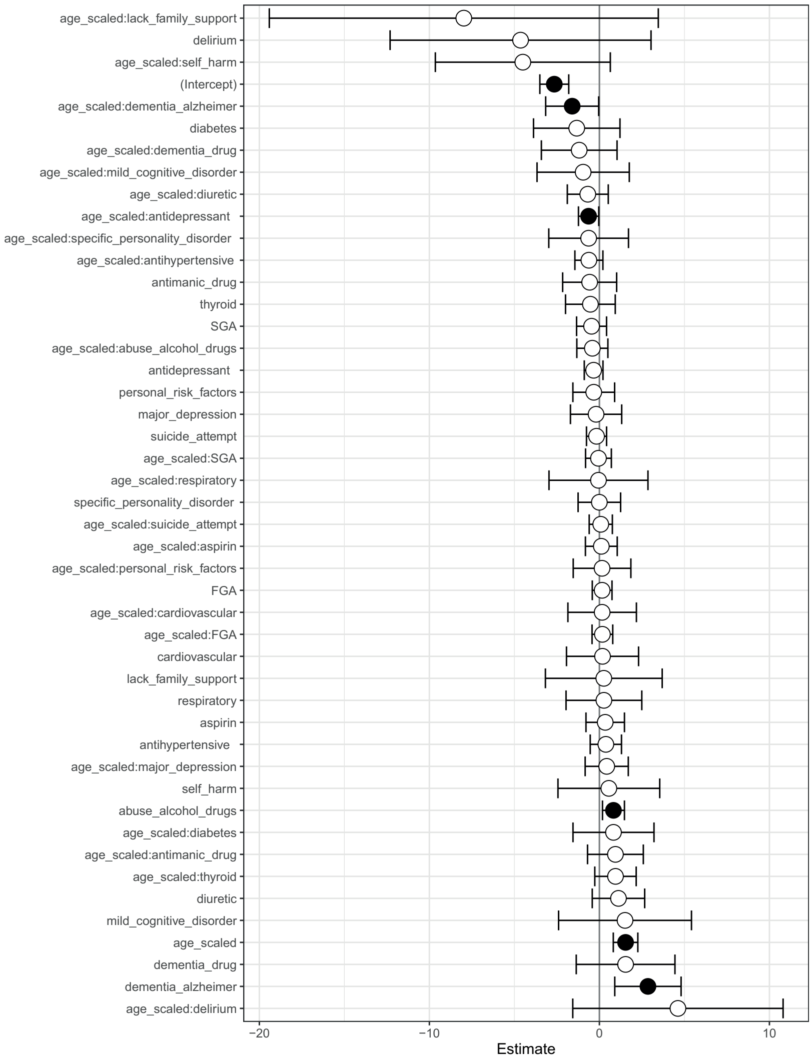

Supplementary Figure 3. Logistic regression model with main effects and all pairwise interactions with age. Log odds ratio for each feature from a logistic regression model for predicting mortality in patients with schizophrenia. The logistic regression model has main effects and all pairwise interactions with age. Shown are confidence intervals and statistical significance (filled dark circles: p-value < 0.05, open circles: not significant).
